# A curated gene and biological system annotation of adverse outcome pathways related to human health

**DOI:** 10.1038/s41597-023-02321-w

**Published:** 2023-06-24

**Authors:** Laura Aliisa Saarimäki, Michele Fratello, Alisa Pavel, Seela Korpilähde, Jenni Leppänen, Angela Serra, Dario Greco

**Affiliations:** 1 Finnish Hub for Development and Validation of Integrated Approaches (FHAIVE), Faculty of Medicine and Health Technology, Tampere University, Tampere, Finland; 2 Division of Pharmaceutical Biosciences, Faculty of Pharmacy, University of Helsinki, Helsinki, Finland; 3 Institute for Advanced Study, Tampere University, Tampere, Finland

**Keywords:** Data integration, Toxicology

## Abstract

Adverse outcome pathways (AOPs) are emerging as a central framework in modern toxicology and other fields in biomedicine. They serve as an extension of pathway-based concepts by depicting biological mechanisms as causally linked sequences of key events (KEs) from a molecular initiating event (MIE) to an adverse outcome. AOPs guide the use and development of new approach methodologies (NAMs) aimed at reducing animal experimentation. While AOPs model the systemic mechanisms at various levels of biological organisation, toxicogenomics provides the means to study the molecular mechanisms of chemical exposures. Systematic integration of these two concepts would improve the application of AOP-based knowledge while also supporting the interpretation of complex omics data. Hence, we established this link through rigorous curation of molecular annotations for the KEs of human relevant AOPs. We further expanded and consolidated the annotations of the biological context of KEs. These curated annotations pave the way to embed AOPs in molecular data interpretation, facilitating the emergence of new knowledge in biomedicine.

## Background & Summary

Adverse outcome pathways (AOPs) are multi-scale models of biological mechanisms connecting molecular interactions between chemical exposures and biological systems (molecular initiating event, MIE) with adverse outcomes (AO) through key events (KE)^1^. KEs are measurable events described at increasing levels of biological complexity and connected through key event relationships (KER) that provide context and justification for the connection between the KEs. The AOP framework is central in modern toxicology, where efforts of shifting towards mechanistic models and alternatives to animal experimentation are taking place. AOPs can guide the development of new approach methodologies (NAMs) which include *in vitro* tests, targeted assays, and prioritisation strategies, and aim to fill the gaps in decision making in chemical risk assessment while also reducing the use of animal experimentation^2^. Similarly, AOPs can be applied to depict mechanisms of disease progression and other biological events^3,4^. AOPs not only provide a convenient framework to represent and interpret biology, but they also help to identify knowledge gaps and support the implementation of novel applications in biomedical research.

While AOPs model the cascade of events from a MIE to an AO at the level of tissues, organs, individuals or even populations, molecular mechanisms of chemical exposures can be investigated through toxicogenomics^5–7^. Toxicogenomics provides a complementary approach to the traditional observation of phenotypic effects of chemical exposures by focusing on the mechanism of action (MOA) of chemicals using omics technologies. This further enables an array of data-driven and computational approaches, including chemical grouping, read-across, and predictive models, and helps to explain why and how an exposure induces its effects^8^. This way, toxicogenomics can also inform the development of novel AOPs and support the application of AOP-based knowledge in the development of NAMs^9–14^. While to date the link between patterns of molecular alteration and AOPs has been investigated at the level of individual or selected AOPs^13–16^, a systematic framework to integrate these two concepts is missing. The primary challenge to this is the lack of thorough and robust annotation that would link biological events to meaningful sets of molecules (genes/proteins/etc.) whose alteration could be monitored through omics technologies. Establishing this link would enable straightforward interpretation of the complex patterns of molecular alteration in a mechanistic way.

AOP-related information is primarily stored in the AOP-Wiki repository (aopwiki.org). Varying levels of annotations (ontologies, taxonomic and life stage applicability, etc.) and metadata are provided to support the use of AOPs. The existing annotations, however, are only suitable to provide general context and associations between concepts, instead of allowing the modelling of the KEs through specific sets of genes. Furthermore, there are inconsistencies in the level of specificity and coverage of the annotations. Previous efforts of annotating KEs through computational approaches have been shown to be successful but they remained at the level of theoretical associations without the intention of modelling the KE-gene relationships^17,18^.

**Fig. 1:**
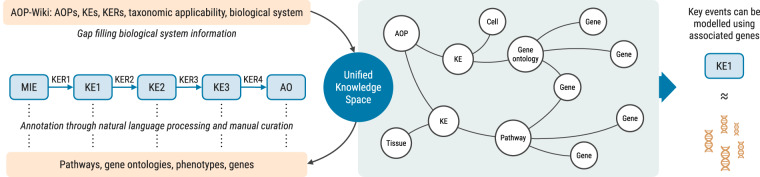
Study overview. Data from the AOP-Wiki was embedded into a previously established knowledge graph, the Unified Knowledge Space (UKS)^24–26^. KEs of human relevant AOPs were annotated to pathways, gene ontology terms, phenotypes, and/or individual genes through natural language processing techniques and manual curation. Furthermore, the existing biological system (organ, tissue, cell type) annotations were amended. The knowledge graph structure was then used to associate genes mapped to the annotated terms to the KEs, allowing KEs to be modelled using sets of genes.

Here, we present a comprehensive annotation of KEs relevant for human health to sets of genes. We integrated techniques of natural language processing (NLP) and manual curation to obtain robust and accurate associations. An initial version of this effort was used in a recent study to build AOP-based NAMs, including experimentally validated *in vitro* biomarkers for pulmonary fibrosis^19^. Furthermore, here we expanded the curation to fill gaps in the biological system annotations provided in the AOP-Wiki for the KEs. This helps to refine the AOPs and supports the reuse of existing KEs in new AOPs, which guides the identification of new links by enhancing the AOP network^14,19–23^. It can further improve applications combining AOPs with physiologically based pharmacokinetic (PBPK) modelling through the addition of relevant cell types, tissues, and organs. The overall strategy of the study is presented in Fig. 1.

## Methods

### Data structure and integration

The previously established knowledge graph, the Unified Knowledge Space (UKS)^19,24–26^ was used as the foundation of the study. The knowledge graph is managed in Neo4j v. 4 (https://neo4j.com/), and the full list of data sources relevant for the present study is listed in Table 1 together with their data retrieval dates, versions, and references.

**Table 1:** Data types and sources.

Data type	Resource	Link	Retrieval date	Version/Release
Pathways	KEGG^34^	https://www.genome.jp/kegg/pathway.html	10/14/2021	Release 100
	WikiPathways^33^	https://www.wikipathways.org/	10/14/2021	Version 20211010
	Reactome^35^	https://reactome.org/	10/9/2021	Version 78
Phenotypes	Human Phenotype Ontology^37^	https://hpo.jax.org/app/	10/14/2021	Release 2021-10-10
	KEGG disease^34^	https://www.genome.jp/kegg/disease/	10/14/2021	Release 100
Gene ontologies	Gene Ontology^36,40^	http://geneontology.org/	10/7/2021	Release 2021-09-01
Genes, gene products	Ensembl^41^	https://www.ensembl.org/index.html	10/31/2019	Release 98
AOPs	Aop-Wiki	https://aopwiki.org/aops.json	10/26/2022	Release 2.5
KEs, KE level, biological system	Aop-Wiki	https://aopwiki.org/events.json	10/26/2022	Release 2.5
KERs	Aop-Wiki	https://aopwiki.org/downloads/aop_ke_ker.tsv	10/26/2022	Release 2.5

Pathways, gene ontology terms (GO), and phenotypes (together referred to as the *gene sets*) were introduced into the UKS as individual nodes with each term corresponding to a single node. Genes associated with pathways and phenotypes were linked to them based on the data from each corresponding database, and the connections between gene ontologies (biological process, molecular function, cellular component) and genes were obtained from GO and Panther (Table 1). All genes were expressed in Ensembl gene identifiers for improved interoperability. AOP-related data were downloaded from the AOP-Wiki through the available API or through separate download files (Table 1) originally in November 2020 and updated in August 2022. AOPs were introduced into the UKS as individual nodes with connections to their associated KEs. Given the same KE can exist under multiple AOPs with distinct KERs, “Specific Key Event” (KE in the context of a specific AOP) nodes were added as descendant nodes of KEs. Labels such as “Molecular Initiating Event” and “Adverse Outcome” were assigned to the *Specific Key Event* nodes where applicable.

### Annotation of key events to gene sets

**Fig. 2:**
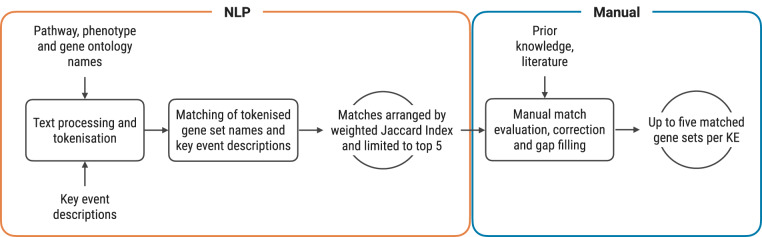
Annotation strategy applied to link gene sets to KEs. Input to the pipeline is marked as text without an outline, process is outlined with a rectangular box, and output is marked by a circular outline. Orange outline indicates steps included in the natural language processing step, while blue outline marks manual curation.

KEs of AOPs relevant for human health risk assessment were annotated to gene sets through a multi-step procedure that combines NLP techniques with manual curation. The outline of the process is summarised in Fig. 2. An AOP was deemed relevant for human health if the reported taxonomic applicability included one or more of the following: *Vertebrata, Mammalia, Catarrhini, Rodentia, Homo sapiens, Rattus Norvegicus, Mus musculus*. The rodent species were included due to their important role as a model organism in human health risk assessment. AOPs with missing information of taxonomic applicability were manually evaluated based on the metadata provided in the corresponding AOP page and included if the pathway was biologically plausible for the selected organisms.

Initial matching and match prioritisation between KEs and pathways, phenotypes, and/or GO terms was performed using NLP techniques. The pipeline was established in Python version 3.7 using the packages nltk^27^ version 3.6.7 and pandas^28,29^ version 1.3.5. The KE descriptions and gene set names as expressed in the MSigDB^30^ (collections H, C2 and C5) were converted to lower case and punctuations were removed. Further text processing included the replacement of concepts consisting of multiple words with one-word concepts using a custom dictionary m. For instance, word pair “positive regulation” was replaced by “upregulation”. The preprocessed text was then split into tokens to be processed individually using the *word_tokenize* function. Tokens corresponding to common words that could lead to spurious matches (e.g., articles and prepositions), were detected and discarded using the list stop words provided by nltk. Finally, different declinations of the same concepts were mapped to the root terms using the *WordNetLemmatizer* available in nltk that makes use of WordNet’s morphological modifications. This included the conversion of plurals into singular forms, different verb tenses into the basic form, as well as the standardisation of different spelling formats (e.g., “pparα” and “pparalpha” map both to “ppar-alpha”). As a result, each KE description and gene set name was presented as a set of tokens, e.g. {“upregulation”, “ppar-alpha”}. Considering that the tokens appear in the KE descriptions and gene set names in varying frequencies, the informative value of each token is not equal. Rare tokens were considered more informative than the common tokens, hence, each token was weighted by its inverse document frequency (IDF)^31^, idf(t) = log(N/d), where N is the total number of gene sets considered and d is the number of gene sets that contain the token t. This means, that the weight of the token is inversely proportional to the number of gene set names and descriptions containing the token. These weights were then applied in the calculation of the weighted Jaccard Index (JIW)^32^ between the sets of tokens x and y of each KE and gene set and used for the matching, $J{I}_{w}\left(x,y\right)=\frac{{\sum }_{i}\min \left({x}_{i},{y}_{i}\right)}{{\sum }_{i}\max \left({x}_{i},{y}_{i}\right)}$. Hence, rare tokens shared by KE descriptions and gene set names leads to a higher matching score than common tokens, making the results more specific. The matches were organised based on the JIW in descending order, and the top five matches were retained. Including up to five annotations for each KE allowed improved specificity and contextualisation when a single gene set would not result in a comprehensive match while also keeping the number of annotations manageable.

The prioritised matches were then manually evaluated and consolidated. This included the individual evaluation of all the matches for their accuracy and correct context, removal of irrelevant matches, and the refinement and gap filling. If the computationally prioritised matches were not biologically relevant or in the correct context, relevant gene sets were manually searched and added from the selected databases (WikiPathways^33^, KEGG^34^, Reactome^35^, GO^36^, Human Phenotype Ontology (HPO)^37^). At this stage, NLP-based matches derived from any other database than the ones listed here were discarded due to limited representation. Given the goal of linking toxicogenomics data to the KEs/AOPs, the gene sets for KEs describing the alteration of an individual gene or gene product were linked to the main functions of the molecule (e.g., the activation of a specific gene or protein to the signalling pathway it drives instead of the individual gene), as such a signal is more likely to be captured from omics data than the specific induction of the gene (product) itself. However, if no distinct signalling pathways or key functions could be identified at the level of the gene sets, the KE was linked to the specific gene itself. If no biologically relevant matches could be identified, the KE remained unannotated. The hierarchical structure of the GO terms was exploited to add specificity to the gene sets by adding the relevant descendants for parent terms when applicable. For example, KE 1457 titled “Induction, Epithelial Mesenchymal Transition” was assigned the following GO terms: GO:0001837 - Epithelial to mesenchymal transition, GO:0010717 - Regulation of epithelial to mesenchymal transition, and GO:0010718 - Positive regulation of epithelial to mesenchymal transition.

The gene set names were mapped to the gene set identifiers and the results of the curation were integrated into the UKS as relationships between the KE nodes and gene set nodes. The level of the annotation (up to five annotations were provided to each KE) was included as an attribute of the edge, allowing future filtering based on the level. After establishing the links between KEs and gene sets, each KE can be represented as the union of all the genes associated to its matched pathways, GO terms and/or phenotypes. For this, human genes associated to each term were retrieved through the UKS.

### Refinement of the biological system annotations

As part of the annotations in the AOP-Wiki, the level (molecular, cell, tissue, organ, individual, population) of the KEs is provided. Similarly, KEs are associated with a biological system that expresses the biological “location” of the KE. However, the provided locations may be limited to the context of the AOP in which the KE was first described. This can result in the duplication of KEs (i.e., the same event is added to the AOP-Wiki as a distinct KE resulting in the loss of the potential connection in a complete AOP network). Furthermore, this data is fully missing for some of the KEs. Completion of this information could improve network-based approaches to AOP research and the development of new AOPs. Hence, the existing biological system annotations were manually evaluated, refined, and extended to include plausible biological systems beyond the originally defined ones. Furthermore, gaps in the annotation were addressed and the already existing cell, tissue, and organ terms were amended with a system level annotation (e.g., respiratory system, endocrine system, etc.) and a cell component annotation, where applicable. For example, if a KE was annotated to a cell term “hepatocyte” in the AOP-Wiki, this annotation was supplemented with an organ/tissue term annotation “liver” and system terms “digestive system, exocrine system, endocrine system”.

The annotations were assigned based on the primary location or context of the KE, as suggested in the AOP-Wiki. However, in cases where an organ or cell term was assigned in the AOP-Wiki, but other organs/tissues or cells were also determined as applicable, the original terms were replaced or amended with other possible organs, tissues, or cell types, with the options separated by “/”. The KE descriptions (names) were used as the primary source of annotation, followed by the metadata provided for the KEs in AOP-Wiki. If the biological system was not clear based on the provided descriptions, a literature search was performed. For KEs at the molecular level (e.g., changes in the expression of individual genes/proteins), Human Protein Atlas^38^ was used to determine relevant cell types and tissues. If the process was applicable for most or all cell types, “eukaryotic cell” was assigned as cell annotation, and the system and tissue/organ annotations were left unassigned to indicate the applicability of a range of tissues and organs. Furthermore, “eukaryotic cell” was introduced as a secondary annotation in cases, where the KE was specified for a distinct cell type or organ/tissue but would be biologically plausible in other cells, tissues, and systems as well. The secondary annotation was established to distinguish between any cells of a specific system, organ or tissue, and a generic eukaryotic cell. Finally, the systems, organs, tissues, cell types, and cell components were collected to a unified dictionary provided as part of the data collection.

## Data Records

### Data overview

**Fig. 3:**
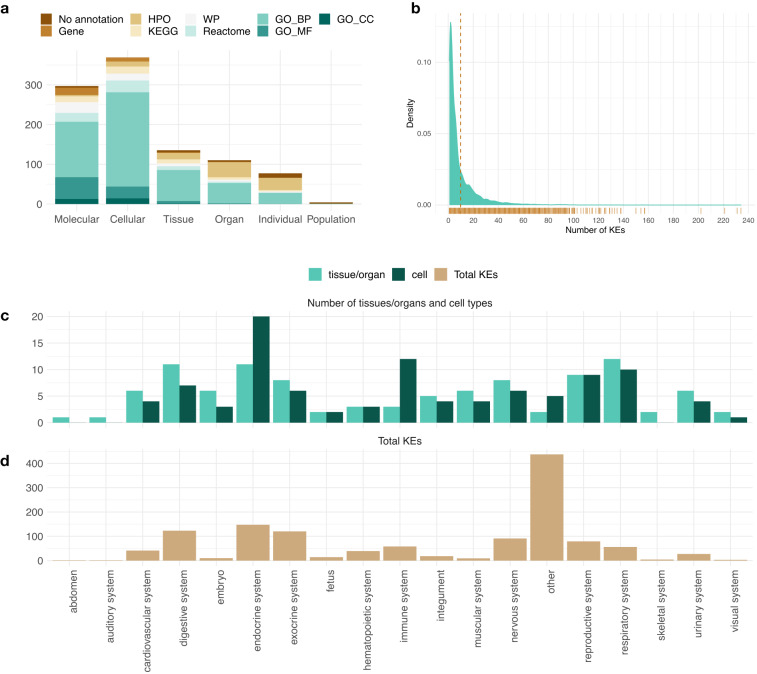
Characteristics of the KE annotation. (**a**) Stacked bar plot representing the proportion of annotation types by KE level. Total height of the bar reflects the number of KEs in each level of biological organisation. Dark brown (no annotation) stack corresponds to the number of KEs with no associated gene sets, while the different annotation types are represented proportionally to their use in each level. HPO = Human Phenotype Ontology, WP = WikiPathways, GO_BP = Gene Ontology Biological Process, GO_CC = Gene Ontology Cellular Component, GO_MF = Gene Ontology Molecular Function. (**b**) The density distribution of the number of KEs associated with each gene. Median of the distribution is indicated with a dashed brown line and the rug below x-axis is used to support the interpretation of the distribution. (**c**) Number of different tissues/organs (turquoise) and cell types (dark green) under each system-level annotation. (**d**) Total number of KEs by system level annotation. The system “other” includes KEs assigned a cell type applicable for a range of tissues and/or systems, and those for which no system could be defined.

The annotated data collection covers 231 AOPs with a total of 997 unique KEs that form 1636 AOP-KE pairs (specific KEs). Of these, 969 unique KEs (1,596 Specific Key Events) were successfully annotated to sets of genes. The number of gene sets associated to KEs ranges from 0 to 5 with a median of 3. Majority of the gene sets represent GO biological processes (total 1,532 annotations), followed by GO molecular function (273), Human Phenotype Ontology (263), Reactome pathways (195), WikiPathways (167), KEGG pathways (154), individual genes (89), and GO cellular components (83). The numbers in brackets correspond to total annotations of each type. The number of KEs at each level of biological organisation together with the proportions of annotation sources by KE level are shown in Fig. 3a. Total number of human-relevant terms present in each data source at the time of data retrieval (information available in Table 1) and their associated genes are summarised in Table 2.

**Table 2:** Number of unique terms and genes used from each data source together with the total amount of human terms and genes present in each source at the time of data retrieval.

Data source	Terms included	Total terms	Genes included	Total genes
GO biological process	746	12380	8817	20411
GO molecular function	158	4434	5252	20878
HPO	171	9946	4233	5209
Reactome	108	2496	6894	12355
WikiPathways	69	701	3108	8808
KEGG	61	334	4097	9454
GO cellular component	49	1754	8434	21809

The number of genes correspond to unique Ensembl gene identifiers.

Each KE is represented as the union of genes linked to its associated gene sets. For instance, Event:1493 “Increased Pro-inflammatory mediators” is represented as all the genes associated to its annotations “GO:0002532 – Production of molecular mediator involved in inflammatory response” and “GO:0006954 – Inflammatory response”. Similarly, each AOP can be represented by the genes linked to its KEs. The number of genes in annotated KEs range from 1 to 6,047 with a median of 82, while the number of genes linked to the AOPs range between 15 and 6,381, (median of 804). In total, the annotations cover 16,825 genes with varying levels of specificity for KEs, i.e., some genes are associated with a large number of distinct KEs, while others are specific to individual KEs. This measure of KE specificity is an important factor in applications focused on the identification of KE specific biomarkers or reporter genes, for example. The distribution of the number of KEs per gene is presented in Fig. 3b.

The biological system annotations were consolidated for all KEs available in the collection. In total, they cover 18 biological systems, 86 specific organs and tissues, and 70 cell types (Fig. 3c,d). Furthermore, 7 distinct cell components were defined. The annotations are provided at varying levels of biological complexity following the specification provided in the AOP-Wiki and the information in the KE description.

### Data files and formats

The data^39^ is available on Zenodo at 10.5281/zenodo.7980953. The provided files as summarised in Table 3.

**Table 3:** Description of files provided as part of the data set.

Data type	Description	File structure	File name
KE to gene set annotation	KE to gene set annotations. Annotations provided by specific KE (AOP-KE pairs).	A spreadsheet file with two sheets, one with annotation provided as the gene set names, one with identifiers. Both sheets contain columns AOP, KE, Specific_KE, Description (KE name), and Match_1 through Match_5.	Gene_set_annotations.xlsx
KE to gene annotation	Direct KE to gene associations. KE associated genes are expressed as the union of all the genes mapped to the gene sets annotated to each KE.	File provided as a tab-separated text file. File contains two columns, one for the KEs and one for the genes. Genes expressed as Ensembl identifiers.	Genes_to_KEs.txt
Gene set identifier to name mapping	Mapping between gene set identifiers and the names used for matching KE descriptions to gene sets. File may be needed if genes are obtained from external sources.	File provided as a tab-separated text file. File contains two columns: term_name and exact_source.	Name_to_ID_mapping.txt
KE to biological system annotation	Annotation of KEs to relevant biological systems at the level of the system, organ/tissue, cell, and cell component.	A spreadsheet with a column for KE name, id, and level, as well as distinct column for each annotation by level, including the secondary annotations, and indication of duplication. Equal annotations are separated by “/”.	Biological_system_annotations.xlsx
Dictionary	A complete listing of all the systems, tissues/organs, cell types, and cell components used in the biological context annotations.	A spreadsheet with five sheets. Complete dictionary covers all combinations of system, organ/tissue, and cell type annotations. Individual dictionaries provide a complete list of systems, organs/tissues, cell types, and cell components.	Dictionary.xlsx

## Technical Validation

While the validity of the gene sets as models of KEs cannot be measures objectively at the scale of this study, we evaluated the consistency and robustness of the KE-gene set annotations by grouping together KEs with Jaccard similarity coefficient (JI) >0.90. In detail, JI was calculated between all pairs of KEs, and transformed into a distance matrix used for clustering. The clustering performed using the *hclust* function from R package stats with the complete method, and the optimal number of clusters was defined so that intra cluster JI was >90. As a result, the KEs grouped into 731 clusters ranging in size from one to 19 KEs, with 128 clusters having at least two (Figure S1). We evaluated the clusters with six or more KEs (total six clusters). The largest cluster was characterised by KEs related to different types of cancer (19 KEs). These KEs were annotated to general pathways in cancer due to the lack of exact gene sets specific for the cancer type, while more specific annotations were available for KEs such as “Liver Cancer” (Event:1395) and “Breast Cancer” (Event:1193), leaving them outside the cluster. The second largest cluster (10 KEs) was formed by all cytotoxicity related KEs, followed by KEs regarding hormone release from the hypothalamus or the anterior pituitary gland (8 KEs), and inflammation (8 KEs). Inflammatory events are covered by a broad range of KEs ranging from different wordings of increased inflammation to more specific inflammatory events, such as “Increased Pro-inflammatory mediators” (Event:1491). The inflammation cluster was formed by the more generic processes, while the specialised KEs either formed a smaller cluster or stood alone. A fertility cluster (8 KEs) was formed by KEs describing decreased fertility and reduced reproductive success. Finally, cell proliferation formed a cluster of 6 KEs. A full list of the clusters is available in Supplementary Table 1 while the JI calculated between each pair of KEs is reported in Supplementary Table 2.

These results reflect the consistency and robustness of the annotations, while also highlighting the differences in ontologies and pathway curations for distinct biological processes. For instance, cancers like breast cancer and hepatocellular carcinoma are well covered and hence KEs of these processes could be assigned specialised gene sets, while liposarcoma and fibrosarcoma could only be matched with more generic pathways in cancer. As the curated pathways and gene ontologies evolve in specificity, the biological context annotations can provide a meaningful tool for refining the KE associated gene sets.

## Usage Notes

The KE-to-gene set annotations presented in this manuscript result from an integrated approach, where computational prioritisation was performed using techniques of natural language processing, and further consolidated by manual curation to ensure appropriate context for the matches. Although this allows the human based assessment of each annotation, it is prone to potential interpretation errors and differences in views of priority and suitability of the matches. Here, the goal was to provide a comprehensive link between the AOP framework and omics data, hence the gene sets associated to KEs and AOPs should accurately reflect each process. While these gene sets do not replace the individual assays targeted for measuring individual KEs, they allow the identification of potential KEs and AOPs from complex molecular data, opening doors to various data-driven applications to AOP development and use. Similarly, the biological context annotations are intended to support the reuse of KEs, and to guide the refinement of the AOP network and the discovery of hidden links between KEs. Although they were curated to accurately reflect the relevant biological locations for the KEs, they may not always include all possible options or exclude those that are not feasible. In practice, the annotations can be used to filter the data and/or the AOP network to only include KEs relevant to a biological system of interest or to merge redundant nodes. This may further result in connections between KEs that were previously not obvious.

These applications were supported by the indication of the gene set similarity as defined by the JI matrix provided (Supplementary Table 2). We previously observed several reasons behind identical gene sets between distinct KEs (JI = 1)^19^. Namely, these include 1) truly duplicated KEs; 2) the same event in different biological systems; 3) subsequent or related KEs mapped to the same terms due to inadequate specificity; and 4) opposite regulation of the same biological event (e.g., increased vs. decreased signaling), where the last case is also due to the lack of specificity in the available gene sets. We believe the consideration of duplicated KEs to be case-dependent. Certain applications may benefit from an approximate grouping based on the similarity of the associated gene sets (e.g., finding functionally related KEs), while others may rely on more robust and accurate refinement (e.g., merging nodes in an AOP network). While the users of these data are encouraged to find an approach that suits their application, the most robust set of duplicates based on semantics, gene set similarity, and the assigned biological context are identified and reported in Biological_system_annotations.xlsx file.

It is also worth noting that AOPs are under constant development, and individual entries are at different phases of completion. Only a handful of the AOPs available in the database are finalised and endorsed (aopwiki.org). This means that the majority of the AOPs and KEs included in this collection are subject to changes. Hence, we suggest the users to refer to the AOP-Wiki (aopwiki.org) for up-to-date information of KE relationships, KE-to-AOP mappings, and any further information that may support the use of this data.

All KE-gene annotations are provided as human gene sets. However, the selected taxonomies also include other species that are often used as model organisms in human health risk assessment. It is worth noting that some of the processes may not be directly applicable to humans. The exact species and the strength of evidence for taxonomic applicability for each AOP can be obtained from the AOP-Wiki. Additionally, the genes associated with the gene sets may differ from those reported in this study depending on the resource used to retrieve the genes. This may be due to the selected gene identifiers, updates in the original databases, as well as differences in the interpretation of hierarchical formats present in the databases (e.g., Gene Ontology). As an example, the GO gene sets used in this study are based on the direct annotations between GO terms and genes, while other resources may include genes annotated to all descendants of the term as well.

## Supplementary information

Supplementary Table 1

Supplementary Table 2

Supplementary Information

## Data Availability

Custom code and data used in the NLP-based prioritisation of the gene set annotations is available in the data repository^39^ on Zenodo at 10.5281/zenodo.7980953 (file aop_mapping_nlp.tar.gz).
