# Supplementary material for: A curated gene and biological system annotation of adverse outcome pathways related to human health
Source: Sci Data. 2023 Jun 24;10:409. doi: 10.1038/s41597-023-02321-w (PMC10290716; doi:10.1038/s41597-023-02321-w)
Supplement: Supplementary file 3 — Supplementary Information [file 41597_2023_2321_MOESM3_ESM.pdf]

A curated gene and biological system annotation of adverse outcome pathways related to human health

Laura Aliisa Saarimäki[1], Michele Fratello[1], Alisa Pavel[1], Seela Korpilähde[1], Jenni Leppänen[1], Angela Serra[1,2] and Dario Greco[1,3,*]

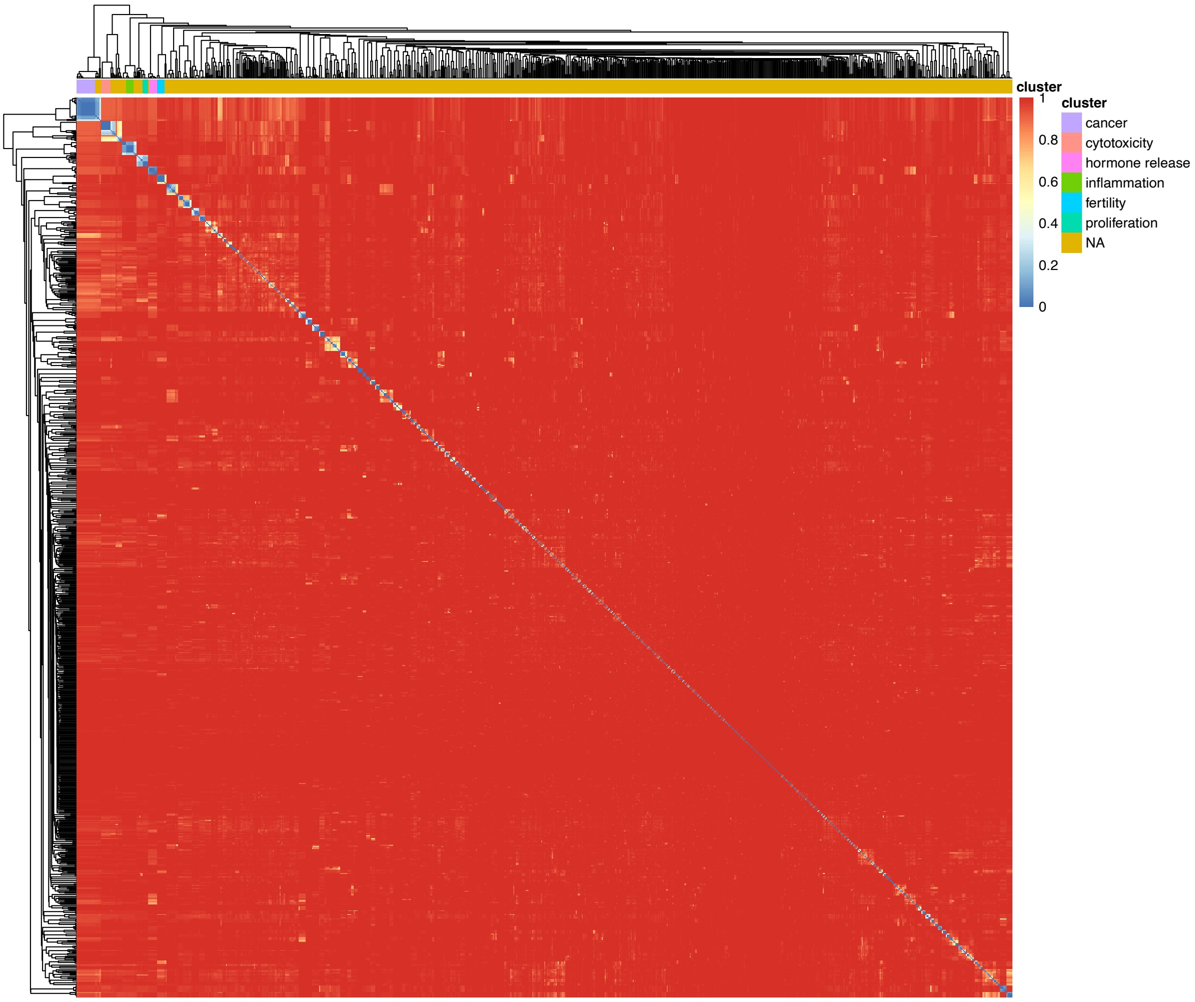

Supplementary Figure 1. Heatmap representation of the KE clustering. The cell colours indicate the distance between pairs of KE, with blue colour corresponding to high similarity while the red colour denotes dissimilarity. Clusters formed by six or more KEs are highlighted by coloured bars on the columns and characterised. NA (yellow) indicates clusters formed by less than five KE that were not characterised in detail.
